# Effect of initial COVID-19 outbreak during first trimester on pregnancy outcome in Wuxi, China

**DOI:** 10.1186/s12884-022-04395-7

**Published:** 2022-01-21

**Authors:** Yun Liu, Ming Dai, Shaidi Tang

**Affiliations:** 1 Department of Obstetrics, The Affiliated Wuxi Maternity and Child Health Care Hospital of Nanjing Medical University, Wuxi, 214000 Jiangsu China; 2 Department of Gastroenterology, Liyang People’s Hospital, Liyang, 213300 Jiangsu China; 3 Department of Education and Science, Liyang People’s Hospital, Liyang, 213300 Jiangsu China

**Keywords:** COVID-19, Disease outbreaks, Pregnancy

## Abstract

**Background:**

A hospital-based retrospective study was conducted to examine the effect of initial COVID-19 outbreak during first trimester on pregnancy outcome in Wuxi, China.

**Methods:**

Women who delivered children at our hospital during June 2020 to July 2020 (control group), and October 2020 to December 2020 (exposure group) were recruited in the present study. All of the participants were not infected with COVID-19. The last menstrual period (LMP) of the exposure group was between January 24th, 2020 and March 12th, 2020, whilst in the control group, the LMP was between May 12th and October 31st, 2019.

**Results:**

There were 1,456 women in the exposure group and 1,816 women in the control group. Women in the exposure group were more susceptible to hypertension during pregnancy (HDP, *P* = 0.004, *OR*[95%*CI*] = 1.90[1.22–2.95]) and gestational diabetes mellitus (GDM, *P* = 0.008, *OR*[95%*CI*] = 1.31[1.08–1.60]) compared to those in the control group. Mothers diagnosed with HDP were more likely to deliver premature infants, leading to a higher rate of low birth weight (all *P* < 0.05). The other common outcomes of pregnancy showed no statistical differences between the two groups.

**Conclusions:**

The initial COVID-19 outbreak might increase the incidence rates of HDP and GDM among pregnant women whose first trimesters were during that period, resulting in higher percentages of premature delivery and low birth weight. These results should be confirmed by studies from other hospitals or cities.

**Supplementary Information:**

The online version contains supplementary material available at 10.1186/s12884-022-04395-7.

## Background

Corona virus disease 2019 (COVID-19) is an illness caused by the Severe Acute Respiratory Syndrome Corona Virus-2. In December 2019, COVID-19 broke out in Wuhan city and spread rapidly to other districts in China. To contain the epidemic, the government immediately activated the highest public health emergency response and imposed a lockdown on January 24th, 2020. During the initial stage of the lockdown, the general public stayed at home and socially isolated themselves to prevent being infected.

In that extraordinary time, confirmed cases increased day by day, whereas health equipment was of shortage and the knowledge of COVID-19 remained insufficient. Consequently, people were prone to experience stress, anxiety and depression [1, 2]. A cross-sectional survey from China indicated that 53.8% of the respondents rated the psychological impact as moderate or severe, and 28.8% reported moderate to severe anxiety symptoms and stress levels [3]. What’s more, the psychological symptoms of females, especially pregnant women, appeared to be more severe than those in the general public [4–6]. Due to the possibility of vertical transmission of the disease, women in the first trimester of pregnancy were vulnerable to high anxiety [7]. For perinatal pregnant women assessed after the declaration of COVID-19 epidemic, they reported higher rates of depressive symptoms than women assessed before the epidemic declaration [8]. Also, these women were more likely to have thoughts of self-harm [8].

However, psychological risk during pregnancy may contribute to several negative consequences, including preterm birth [9] and pre-eclampsia [10]. In terms of pathology, maternal stress tends to release more cortisol [9, 10], and subsequently reduces the lymphocyte sensitivity to glucocorticoids [10]. As steroid resistance is developed, levels of proinflammatory cytokines interleukin (IL)-1β, IL-6 and tumor necrosis factor α are increased, whereas antiinflammatory sytokin IL-10 are decreased [9, 10]. Finally, the dampened immune system leads to an elevated risk of adverse maternal–fetal outcomes. In terms of epidemiology, higher allostatic load in early pregnancy was associated with increasing odds of preeclampsia [11]. Furthermore, women exposed to earthquake during their first trimesters delivered smaller newborns and were more likely diagnosed with preterm delivery and premature rupture of membranes [12].

Pregnancy outcomes might also be affected by the initial stage of the COVID-19 outbreak in Wuxi, China. As the most serious period of the COVID-19 outbreak in Wuxi city was approximately from January to March in 2020, women who became pregnant just during that period had delivered children from October 2020 to December 2020. Therefore, the aim of this study was to examine the effects of COVID-19 outbreak on pregnancy outcomes of these special pregnant women who had delivered children at our hospital.

## Materials and methods

### Ethics approval

The present study was approved by the ethics committee of the Affiliated Wuxi Maternity and Child Health Care Hospital of Nanjing Medical University (No. 2021–06-0615–11). In our database, the patient information of name and telephone number was anonymized.

### Study design, area and period

A hospital-based retrospective study was carried out in Wuxi city, which is about 700 km far from Wuhan city where the first case COVID-19 was diagnosed. The Affiliated Wuxi Maternity and Child Health Care Hospital of Nanjing Medical University is the largest maternal hospital in Wuxi, and was recommended for childbirth by the government at the beginning of the outbreak. The total number of births in the hospital was 10,000 per year.

The level I and II emergency responses in Wuxi city were activated on January 24th, 2020 and February 24th, 2020, respectively, and ended on March 27th, 2020. Hence, the initial stage of COVID-19 pandemic was defined as the period from January 24th, 2020 to March 27th, 2020.

### Participants

**Fig. 1:**
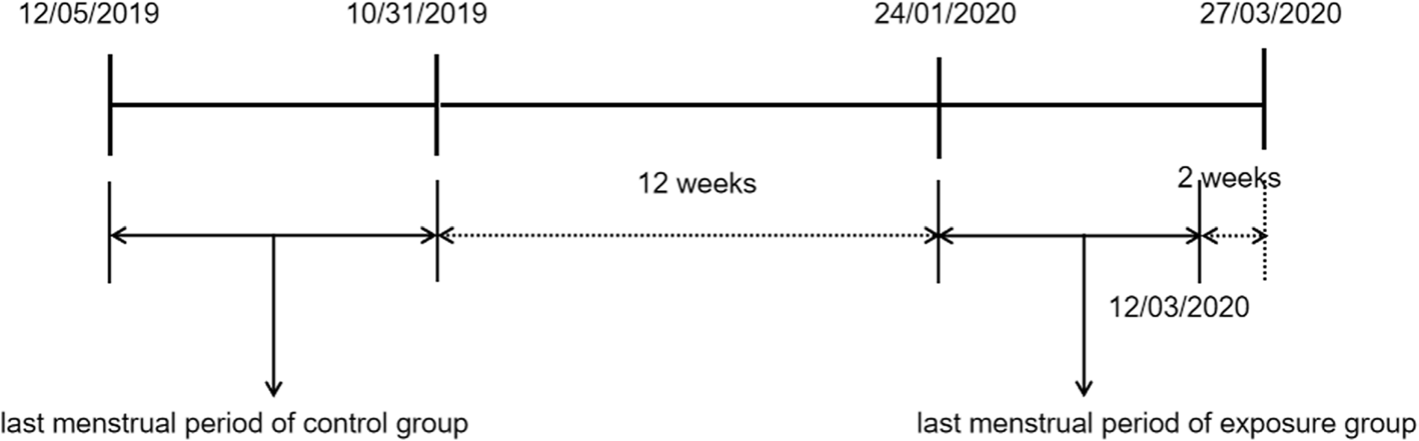
Last menstrual period of two groups

Women who delivered children at our hospital from June 2020 to July 2020 (control group) and October 2020 to December 2020 (exposure group) were recruited to the present study. All of them were free from COVID-19 infection. According to the last menstrual period (LMP), we divided participants into two groups. Exposure group: women became pregnant in the initial stage of COVID-19 pandemic, and at least two weeks of first trimesters overlapped with that period; the LMP was from January 24th, 2020 to March 12th, 2020. Control group: women became pregnant at least 12 weeks before the COVID-19 outbreak; the LMP was from May 12th to October 31st, 2019. Figure 1 showed the exposure group legibly.

### Data collection

We extracted participants’ information from the inpatient medical records and antenatal examination books. The details were as follows.

Demographic characteristics: Age, obesity (BMI of pre-pregnancy ≥ 28), gravida and parity.

Clinical variables: Gestational weeks, multiple pregnancy, history of cesarean delivery and myomectomy, in vitro fertilization (IVF), carriage of hepatitis B virus (HBV), fibroid.

Complications or outcomes of pregnancy: Cesarean, hypertension during pregnancy (HDP), gestational diabetes mellitus (GDM), premature rupture of membranes, pregnancy with hypothyroidism, placental abnormality (including placenta previa, low-lying placenta, placental abruption, cord velamentous insertion, et al.), abnormal amniotic fluid volume (including oligohydramnios and polyhydramnios), pregnancy with thrombocytopenia, intrahepatic cholestasis of pregnancy (ICP), pregnancy with anemia.

Characteristics of fetus or newborns: Gender, intrauterine fetal distress, fetal growth restriction, malposition (all position but not occipitoanterior), premature delivery and low birth weight.

### Statistical analysis

IBM SPSS Statistics 25 (IBM Corp., Armonk, NY, USA) software was used to perform statistical analysis. The significance level (*P*-value) was declared at 0.05. Mean ± standard deviation (*SD*) was applied to describe the continuous data if it met the normal distribution; otherwise, medians and quartiles were applied. Ratios were used to describe the enumeration data. To detect the differences between two groups, *t* test or *Mann–Whitney U* test was applied for the continuous data, depending on the normality; *χ2* test was applied for the continuous data. Finally, a binary non-conditional *logistic* model was applied to conduct multivariate regression analysis to control for potential confounders. The odds ratio (*OR*) and 95% confidence interval (*CI*) were used to assess the effect of potential related factors.

## Results

### General characteristics

From June to July and October to December in 2020, a total of 3,272 pregnant women delivered children at our hospital. Overall, there were 1,456 women in the exposure group and 1,816 women in the control group. Table 1 reported the participants’ demographic and clinical characteristics. Except for gestational weeks and IVF, no statistically significant differences were found between the two groups. Basically, the general characteristics of the two groups were balanced.

**Table 1:** Demographic and clinical characteristics of exposure and control groups

variables		exposure group (*n* = 1,456)	control group (*n* = 1,816)	*P*-value
Age (years)	Mean ± *SD*^a^	29.88 ± 4.08	29.75 ± 4.16	0.365
Obesity (%)	Yes	40 (2.7)	54(3.0)	0.753
Gravida	Median (25%-75%)	2 (1–3)	2 (1–3)	0.452
Parity	Median (25%-75%)	1 (1–2)	1 (1–2)	0.132
Gestational weeks	Mean ± *SD*^a^	38.89 ± 1.73	39.19 ± 1.24	< 0.001^*^
Multiple pregnancy (%)	Yes	20 (1.4)	34 (1.9)	0.266
History of cesarean delivery and myomectomy (%)	Yes	303 (20.8)	347 (19.1)	0.225
In vitro fertilization (%)	Yes	39 (2.7)	107 (5.9)	< 0.001^*^
Carriage of HBV^b^ (%)	Yes	62 (4.3)	54 (3.0)	0.057
Fibroid (%)	Yes	44 (3.0)	78 (4.3)	0.063

^a^*SD* standard deviation, ^b^*HBV* hepatitis B virus

^*^P < 0.05

### Differences in pregnancy outcomes between the two groups

As shown in Table 2, there were no statistical differences of the percentages of cesarean, premature rupture of membranes, pregnancy with hypothyroidism, placental abnormality, abnormal amniotic fluid volume, pregnancy with thrombocytopenia, ICP and pregnancy with anemia (all *P* > 0.05) between the two groups. However, the rates of HDP and GDM were higher in the exposure group compared to the control group (all *P* < 0.05).

**Table 2:** Complications or outcomes of pregnancy between two groups

variables		exposure group (*n* = 1,456)	control group (*n* = 1,816)	*P*-value
Cesarean (%)	Yes	633 (43.5)	852 (46.9)	0.052
HDP^a^ (%)	Yes	52 (3.6)	40 (2.2)	0.019^*^
GDM^b^ (%)	Yes	235 (16.1)	234 (12.9)	0.009^*^
Premature rupture of membranes (%)	Yes	256 (17.6)	290 (16.0)	0.220
Pregnancy with hypothyroidism (%)	Yes	73 (5.0)	117 (6.4)	0.084
Placental abnormality (%)	Yes	50 (3.4)	47 (2.6)	0.177
Abnormal amniotic fluid volume (%)	Yes	142 (9.8)	213 (11.7)	0.079
Pregnancy with thrombocytopenia (%)	Yes	7 (0.5)	13 (0.7)	0.500
ICP^c^ (%)	Yes	18 (1.2)	11 (0.6)	0.062
Pregnancy with anemia (%)	Yes	26 (1.8)	35 (1.9)	0.796

^a^*HDP* hypertension during pregnancy, ^b^*GDM* gestational diabetes mellitus, ^c^*ICP* intrahepatic cholestasis of pregnancy

^*^P < 0.05

### Differences of characteristics of fetuses or newborns between two groups

As shown in Table 3, there were no statistical differences of gender of newborns, intrauterine fetal distress, fetal growth restriction and malposition between the two groups (all *P* > 0.05). Nevertheless, increased percentages of premature delivery and low birth weight were showed in the exposure group (all *P* < 0.05).

**Table 3:** Characteristics of fetuses or newborns between two groups

variables		exposure group (*n* = 1,456)	control group (*n* = 1,816)	*P*-value
Gender (%)	Male	751 (51.6)	969 (53.4)	0.324
Intrauterine fetal distress (%)	Yes	8 (0.5)	5 (0.3)	0.267
Fetal growth restriction (%)	Yes	11 (0.8)	11 (0.6)	0.669
Malposition (%)	Yes	74 (5.1)	80 (4.4)	0.406
Premature delivery(%)	Yes	125 (8.6)	105 (5.8)	0.002^*^
Low birth weight (%)	Yes	70 (4.8)	49 (2.7)	0.001^*^

^a^*SD* standard deviation

^*^P < 0.05

### Influence of COVID-19 pandemic on HDP and GDM

To estimate the adjusted *ORs* of the effect of COVID-19 pandemic on HDP and GDM, we conducted multivariate *logistic* regression analyses, adjusting all potential factors. As shown in Table 4, women whose first trimesters were during the initial stage of the COVID-19 outbreak, were more susceptible to HDP (*P* = 0.004, *OR*[95%*CI*] = 1.90[1.22–2.95]) and GDM (*P* = 0.008, *OR*[95%*CI*] = 1.31[1.08–1.60]) than those in the control group.

**Table 4:** Adjusted *ORs* of the effect of COVID-19 pandemic on HDP^a^ and GDM^b^

Variables	Adjusted variables	*P*-value	Adjusted *OR*^c^	95%*CI*^d^
HDP^b^	Age, obesity, multiple pregnancy, history of cesarean delivery and myomectomy,gravida, parity, IVF^e^, carriage of HBV^f^, fibroid	0.004^*^	1.90	1.22–2.95
GDM^a^		0.008^*^	1.31	1.08–1.60

^a^*HDP* hypertension during pregnancy, ^b^*GDM* gestational diabetes mellitus, ^c^*OR* odds ratio, ^d^*CI* confidence interval, ^e^*IVF* in vitro fertilization, ^f^*HBV* hepatitis B virus

^*^P < 0.05

### Distribution of HDP types between two groups

Table 5 present the details of the HDP of the two groups whilst no difference was found in the distribution of the HDP types.

**Table 5:** Distribution of HDP^a^ types between two groups

HDP^a^ types	exposure group (*n* = 52)	Control group (*n* = 40)	*P*-value
Gastational hypertension	18 (34.6)	14 (35.0)	0.885
Pre-eclampsia	18 (34.6)	10 (25.0)	
Severe pre-eclampsia	8 (15.4)	8 (20.0)	
Chronic hypertension complicating pre-eclampsia	4 (7.7)	4 (10.0)	
Pregnancy with chronic hypertension	4 (7.7)	4 (10.0)	

^a^*HDP* hypertension during pregnancy

### Associations of HDP and GDM with premature and low birth weight

As premature delivery and low birth weight might be not directly associated with the exposure of COVID-19 outbreak, we conducted *χ2* tests to explore the associations with HDP and GDM, respectively. Table 6 showed that, mothers diagnosed with HDP were more likely to deliver premature infants, leading to a higher rate of low birth weight (all *P* < 0.05). However, GDM did not affect the percentages of both premature delivery and low birth weight (all *P* > 0.05).

**Table 6:** Associations of HDP and GDM with premature infant and low birth weight

outcomes		HDP^a^		GDM^b^	
		Yes	No	Yes	No
Premature delivery (%)	Yes	29 (12.6)	201 (87.4)	34 (14.8)	196 (85.2)
	No	63 (2.1)	2,979 (97.9)	435 (14.3)	2,607 (85.7)
	*P*-value	< 0.001^*^		0.845	
Low birth weight (%)	Yes	16 (13.4)	103 (86.6)	13 (10.9)	106 (89.1)
	No	76 (2.4)	3,077 (97.6)	456 (14.5)	2,697 (85.5)
	*P*-value	< 0.001^*^		0.292	

^a^*HDP* hypertension during pregnancy, ^b^*GDM* gestational diabetes mellitus

^*^P < 0.05

## Discussion

In the current study, we suggested that the COVID-19 outbreak seemed to have a negative impact on the outcomes among pregnant women, since the percentages of GDM, HDP, premature delivery and low birth weight were higher in the exposure group. However, we didn’t find any statistical differences between the exposure and control groups in complications or outcomes of (1) pregnant women, including cesarean, premature rupture of membranes, pregnancy with hypothyroidism, placental abnormality, abnormal amniotic fluid volume, pregnancy with thrombocytopenia, ICP and pregnancy with anemia; (2) fetuses or newborns, including gender of newborns, intrauterine fetal distress, fetal growth restriction and malposition. In addition, no difference was found in the distribution of HDP types between the two groups.

Pandemic of infectious diseases or natural disasters always cause various, indirect effects on public health, especially for pregnant women. It had been noted that women exposed to the 1918 Spanish flu while pregnant showed higher rates of premature delivery in the short term [13]. In addition, women exposed to the 2010 Chilean earthquake during the first trimester had higher risks of smaller newborns, preterm deliveries and premature rupture of membranes, comparing to those exposed at third trimester [12]. For the Calgary 2013 flood, there was a small increase in new cases of gestational hypertension but not other health outcomes in flood affected areas [14]. In the present survey, we indicated the similar results with the studies mentioned above. Women whose first trimesters exposed to the initial stage of COVID-19 outbreak were more prone to be diagnosed with GDM and HDP. Furthermore, according to Table 6, HDP might lead to increased risks of premature delivery and low birth weight.

For the increased rates of HDP and GDM during the COVID-19 lockdown, the reasons haven’t been fully understood. Based on the available references, we supposed that the mental illnesses and altered lifestyles might cause these observations. On one hand, as has been stated in the Introduction part, psychological risk may dampen the immune system and could potentially lead to HDP [9–11]. On the other hand, the altered lifestyles during lockdown might contribute to the elevated percentages of HDP and GDM. Firstly, people’s physical activities were reduced due to the home confinement. It has been widely established that reduced physical activity during pregnancy may increase pre-eclampsia and GDM risks [15–17]. Secondly, a number of people changed the dietary habits and started eating and snacking more [18], probably resulting from the anxiety caused by COVID-19[19]. However, dietary pattern characterized by salty snack has been demonstrated as a risk of HDP and GDM [17, 20]. Finally, the excessive use of smart electronic products should be drawn attention to. In the time of lockdown, people tended to spend more time on using the smart products with electronic screen for entertainment, social communications, shopping, work, studying and so on. Our previous research found a significant relationship between HDP and electronic screen exposure before nocturnal sleep [21]. Also, usage of smartphone only for entertainment was also a risk factor for HDP [21]. Hence, we assumed that excessive exposure to electronic screen during the COVID-19 lockdown had a negative effect on the susceptibility of HDP.

Limitations were also existed in our study. Above all, as one of pregnant outcomes, we did not take the early abortion into consideration. Then, we did not collect the mental health parameters of participants. In that special time, all of the hospital staff were busy doing clinical work and had no time to collect the data. Finally, our results were from a single center, so it couldn’t represent other districts in China, especially in the Hubei province.

## Conclusions

In conclusion, the initial stage of COVID-19 outbreak might increase the incidence rates of HDP and GDM among pregnant women whose first trimesters were during that period, resulting in higher percentages of premature delivery and low birth weight. These results should be confirmed by studies from other hospitals or cities. Nevertheless, as the infection control measures were still there, we suggest pregnant women should maintain healthy diets and physical activity in the early stages of pregnancy.

## Supplementary Information

**Additional file 1.**

## Data Availability

The datasets used and/or analyzed during the current study are available from the corresponding author on reasonable request.

## Abbreviations

COVID-19: Corona virus disease 2019
IL: Interleukin
IVF: In vitro fertilization
HBV: Hepatitis B virus
GDM: Gestational diabetes mellitus
HDP: Hypertension during pregnancy
ICP: Intrahepatic cholestasis of pregnancy
SD: Standard deviation
OR: Odds ratio
CI: Confidence interval
